# Comparative Infections of Zika, Dengue, and Yellow Fever Viruses in Human Cytotrophoblast-Derived Cells Suggest a Gating Role for the Cytotrophoblast in Zika Virus Placental Invasion

**DOI:** 10.1128/spectrum.00630-23

**Published:** 2023-05-25

**Authors:** Mercedes Viettri, Gerson Caraballo, Maria Elena Sanchez, Aurora Espejel-Nuñez, Abigail Betanzos, Vianney Ortiz-Navarrete, Guadalupe Estrada-Gutierrez, Porfirio Nava, Juan E. Ludert

**Affiliations:** a Department of Infectomics and Molecular Pathogenesis, Center for Research and Advanced Studies (CINVESTAV-IPN), Mexico City, Mexico; b Research Division, National Institute of Perinatology, Mexico City, Mexico; c Department of Molecular Biomedicine, Center for Research and Advanced Studies (CINVESTAV-IPN), Mexico City, Mexico; d Department of Biophysical Physiology and Neuroscience, Center for Research and Advanced Studies (CINVESTAV-IPN), Mexico City, Mexico; Regional Centre for Biotechnology

**Keywords:** Zika virus, dengue virus, yellow fever virus vaccine, HTR8 cells, U937 cells, cytotrophoblast

## Abstract

The Zika virus (ZIKV) is teratogenic and considered a TORCH pathogen (toxoplasmosis [Toxoplasma gondii], rubella, cytomegalovirus, herpes simplex virus [HSV], and other microorganisms capable of crossing the blood-placenta barrier). In contrast, the related flavivirus dengue virus (DENV) and the attenuated yellow fever virus vaccine strain (YFV-17D) are not. Understanding the mechanisms used by ZIKV to cross the placenta is necessary. In this work, parallel infections with ZIKV of African and Asian lineages, DENV, and YFV-17D were compared for kinetics and growth efficiency, activation of mTOR pathways, and cytokine secretion profile using cytotrophoblast-derived HTR8 cells and monocytic U937 cells differentiated to M2 macrophages. In HTR8 cells, ZIKV replication, especially the African strain, was significantly more efficient and faster than DENV or YFV-17D. In macrophages, ZIKV replication was also more efficient, although differences between strains were reduced. Greater activation of the mTORC1 and mTORC2 pathways in HTR8 cells infected with ZIKV than with DENV or YFV-17D was observed. HTR8 cells treated with mTOR inhibitors showed a 20-fold reduction in ZIKV yield, versus 5- and 3.5-fold reductions for DENV and YFV-17D, respectively. Finally, infection with ZIKV, but not DENV or YFV-17D, efficiently inhibited the interferon (IFN) and chemoattractant responses in both cell lines. These results suggest a gating role for the cytotrophoblast cells in favoring entry of ZIKV, but not DENV and YFV-17D, into the placental stroma.

**IMPORTANCE** Zika virus acquisition during pregnancy is associated with severe fetal damage. The Zika virus is related to dengue virus and yellow fever virus, yet fetal damage has not been related to dengue or inadvertent vaccination for yellow fever during pregnancy. Mechanisms used by the Zika virus to cross the placenta need to be deciphered. By comparing parallel infections of Zika virus strains belonging to the African and Asian lineages, dengue virus, and the yellow fever vaccine virus strain YFV-17D in placenta-derived cytotrophoblast cells and differentiated macrophages, evidence was found that Zika virus infections, especially by the African strains, were more efficient in cytotrophoblast cells than dengue virus or yellow fever vaccine virus strain infections. Meanwhile, no significant differences were observed in macrophages. Robust activation of the mTOR signaling pathways and inhibition of the IFN and chemoattractant response appear to be related to the better growth capacity of the Zika viruses in the cytotrophoblast-derived cells.

## INTRODUCTION

Zika virus (ZIKV) is a member of the genus *Flavivirus*, mainly transmitted by mosquitoes (1, 2). Although first isolated in 1947 in the Zika forests in Uganda, the first human outbreak was reported in 2007 on Yap Island. In May 2015, the first cases in Brazil were reported, and by 2016, ZIKV infections were reported by most countries in the Americas (1, 3). ZIKV is classified into two lineages, African and Asian, with the Asian lineage responsible for all of the outbreaks in the Americas so far (4).

ZIKV crosses the blood-placenta barrier, causing miscarriages and congenital Zika syndrome (CZS), characterized by neurological disorders (microcephaly, anencephaly, and hydrocephalus), eye anomalies, and congenital contractures (5, 6). Therefore, ZIKV is classified as a TORCH pathogen (toxoplasmosis [Toxoplasma gondii], rubella, cytomegalovirus, herpes simplex virus [HSV], and other microorganisms capable of crossing the blood-placental barrier) (5). In contrast, other mosquito-borne, phylogenetically, and epidemiologically related flaviviruses, such as dengue virus (DENV) or the attenuated vaccine strain of yellow fever virus (YFV-17D) are not teratogenic. Although the presence of dengue virus-infected macrophages in the placenta and umbilical cords has been documented (7–9), acquisition of dengue virus infection during pregnancy seems to be related to having effects on the mother and sometimes miscarriages (10–12), but not to teratogenic effects. Likewise, evidence exists indicating that the inadvertent application of the 17D yellow fever vaccines during early pregnancy causes no harm to the fetus (13, 14).

The main barrier of defense to avoid the vertical transmission of pathogens at the maternal-fetal interface is the chorionic villi, floating in close contact with the maternal blood in the intervillous space (15, 16). Chorionic villi are covered by two cell layers: the syncytiotrophoblast (STB) outer layer, formed from syncytialized trophoblasts in their terminal phase of differentiation, and the cytotrophoblast (CTB), an internal layer made up of trophoblasts that are still in the differentiation phase. These cell layers protect the placental stroma, where fibroblasts, Hofbauer cells (HBCs), and fetal capillaries are found (15, 16). The STB is refractory to ZIKV infection due to the secretion of lambda interferon (IFN-λ) (17). The rest of the villous cells, including trophoblasts and especially HBCs, are susceptible to ZIKV infection (18–20).

The mechanisms used by ZIKV to cross the blood-placenta barrier are not fully understood and need to be deciphered. One model proposes that during the first trimester of pregnancy, invasive trophoblasts encounter ZIKV-infected maternal cells of the basal decidua. Other routes proposed are that infected trophoblasts spread the virus from the decidua parietal across the amniochorionic membranes and release the virions directly into the amniotic fluid (21), or the entry of ZIKV is facilitated by cross-reactive DENV maternal antibodies through transcytosis (22). Additionally, paracellular routes of invasion have also been suggested (23).

Regardless of the route used by ZIKV to enter the placental stroma, CTB cells ought to play a role in the establishment of the infection in the microvillous core. A previous work reported that CTB-derived HTR8 cells were equally susceptible to ZIKV, DENV, and YFV-17D infections, yet ZIKV was found to induce a lower IFN response and a higher proinflammatory response than DENV or YFV (24). In this work, two ZIKV strains, of African and Asian lineages, a DENV strain, and the YFV-17D vaccine strain were again compared side by side for their growth capacity in HTR8 cells and cytokine and chemokine responses. In addition, the capacity of those viruses to activate the mTOR pathways in HTR8 cells was also measured. Our results indicate that CTBs are significantly more susceptible to ZIKV infection, especially by African lineage strains, than to DENV and YFV-17D. ZIKV strains also showed a higher capacity to activate the mTOR pathways and to suppress the IFN response in these cells than DENV and YFV-17D, thus, providing a likely mechanistic explanation for these observations. In contrast, M2 differentiated human macrophages (M2-Mϕs) showed little differences in their susceptible to the 4 strains tested. These results shed new light on the mechanisms used by ZIKV to reach the placental stroma.

## RESULTS

### Infection efficiency of ZIKV, DENV, and the YFV-17D vaccine strain in HTR8 cells and M2 differentiated macrophages.

**FIG 1:**
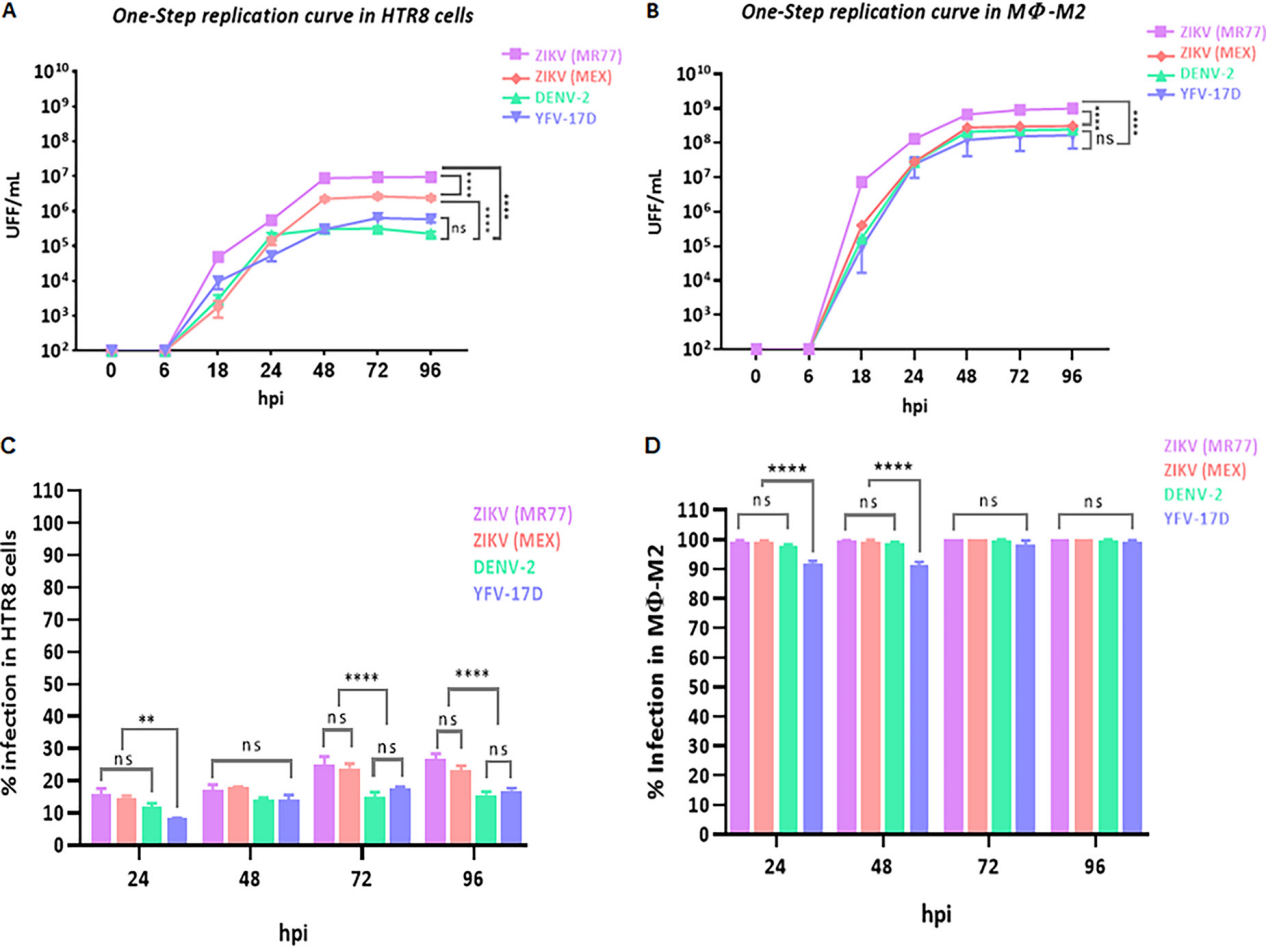
Replication efficiency of ZIKV-MR77, ZIKV-MEX, DENV-2, and YFV-17D vaccine strains in HTR8 cells and differentiated macrophages. (A and B) One-step growth curves in HTR8 cells (A) and M2 differentiated macrophages (Mϕ) derived from U937 cells (B). Infections (MOI = 3) were synchronized by incubation of the monolayers on ice. Supernatants were titrated in Vero-E6 cells using focus-forming unit (FFU) assays. Curves show mean titers ± standard deviation from at least 3 independent experiments. (C) Percentage of infected HTR8 cells; (D) percentage of infected M2 macrophages. Infected cells were stained using a cross-reactive anti-NS3 MAb, and results were expressed as percentage of positive cells in relation to the total number of cells stained with DAPI. *n* = 3 for both cell lines. ****, *P* ≤ 0.0001; **, *P* ≤ 0.0090; ns, not significant.

To better understand the role played by the CTB in the process of ZIKV placental crossing, the efficiency of infection and replication of two ZIKV strains, belonging to the African and Asian genotypes, was evaluated and compared with those of the DENV and YFV-17D strains, using CTB-derived HTR8 cells. In addition, U937 monocytes differentiated to M2 macrophages (M2-Mϕs) were used as an HBC model for comparison. The M2-Mϕ phenotype was confirmed through expression analysis of the differentiation markers CD14, CD163, and CD209 (25, 26) (see Fig. S1 and S2 in the supplemental material); more than 95% of the culture expressed CD14, CD163, and CD209, indicating the acquisition of a macrophage M2 phenotype. One-step growth curves showed that HTR8 cells were susceptible to all of the strains tested; however, they were significantly more susceptible to ZIKV strains. By 48 h postinfection (hpi), the virus progeny for the ZIKV-MR77 and ZIKV-MEX strains reached titers significantly higher than those for DENV and YFV-17D strains; 1 × 10^7^ and 1.5 × 10^6^ focus-forming units (FFU)/mL were obtained for the ZIKV-MR77 and MEX strains, respectively, which are more than 1 log higher than the titers reached for DENV and the YFV-17D vaccine strains (1 × 10^5^ FFU/mL) at the same times (Fig. 1A). On the other hand, M2-Mϕ cells were shown to be highly susceptible to infection by all strains, with titers for ZIKV-MR77 and ZIKV-MEX reaching nearly 1 × 10^9^ and 1 × 10^8^ FFU/mL by 48 hpi. Yet the differences in yield between ZIKV strains and DENV and YFV-17D, although still significant, were reduced to less than 1 log (Fig. 1B).

The higher ZIKV strains’ infection efficiency in HTR8 cells was corroborated when the percentage of infected cells (Fig. 1C) or the amount of virus produced per cell was determined. ZIKV strains always showed a higher percentage of infected HTR8 cells than DENV or YFV-17D, with differences becoming statistically significant (24 to 25% versus 15 to 17%; *P* ≤ 0.0001) at 72 hpi. In turn, macrophages were infected over 90% by all strains even at 24 hpi (Fig. 1D). Virus yield per cell was calculated, with the data obtained from the viral replication kinetics and the infection percentages (Table 1). Infected HTR8 cells produced up to 20-fold times more ZIKV-MR77 viruses than DENV- or YFV-17D-infected cells (275 versus 12 and 14, respectively; *P* ≤ 0.0001). For the ZIKV-MEX strain, differences in virus yield per cell with DENV-2 and YFD-17D strains were less marked, reaching 5-fold at 48 hpi, but were still significant. Finally, macrophages were significantly more permissive for ZIKV-MR77 than for the other strains, with 5-fold differences for ZIKV-MEX, DENV-2, and YFV-17D at 48 hpi.

**TABLE 1:** Virus yield per cell in CTB cells and M2 differentiated macrophages infected with ZIKV-MR77, ZIKV-MEX, DENV-2, and the YFV-17D vaccine strain at different times postinfection^*a*^

Cell type and virus	Time (hpi)	VP^*b*^/cell	Fold change vs:			
			ZIKV-MR77	ZIKV-MEX	DENV-2	YFV-17D
CTB cells						
ZIKV-MR77	24	18	1	3.6	2	6
	48	275	1	4.4	22	19.6
	72	194	1	3.4	17.6	10.8
	96	196	1	3.7	28	11.5
ZIKV-MEX	24	5		1	0.5	1.7
	48	63		1	5.3	4.5
	72	57		1	5.2	3.2
	96	53		1	7.6	3.1
DENV-2	24	9			1	3
	48	12			1	0.9
	72	11			1	0.6
	96	7			1	0.4
YFV-17D	24	3				1
	48	14				1
	72	18				1
	96	17				1
M2-Mϕs						
ZIKV-MR77	24	653	1	4.6	4.6	4.9
	48	3,382	1	2.7	3.2	4.4
	72	4,527	1	3.0	3.9	5.4
	96	4,933	1	3.2	4.1	5.7
ZIKV-MEX	24	141		1	1	1.1
	48	1,253		1	1.2	1.6
	72	1,510		1	1.3	1.8
	96	1,543		1	1.3	1.8
DENV-2	24	143			1	1.1
	48	1,055			1	1.4
	72	1,162			1	1.4
	96	1,215			1	1.4
YFV-17D	24	134				1
	48	766				1
	72	846				1
	96	866				1

a Shown are the virus yield per cell in cytotrophoblastic (CTB) cells and M2 differentiated macrophages (M2-Mϕs) infected with ZIKV-MR77 (African lineage), ZIKV-MEX (Asian lineage), DENV-2, and the YFV-17D vaccine strain, calculated at different 24, 48, 72, 96 hpi. Fold differences among viruses are indicated.

b VP viral particles.

Our results indicate that CTB-derived cells and M2-Mϕs are susceptible to infection by ZIKV, DENV, and YFV-17D. However, at variance with Luo et al. (24), our results also indicate that HTR8 cells are significantly more susceptible to ZIKV strains in terms of infection and replication than to DENV and YFV-17D. Although this trend was also observed in M2-Mϕs, the differences between strains were less marked.

### Cocultures of CTB-derived cells and differentiated macrophages.

**FIG 2:**
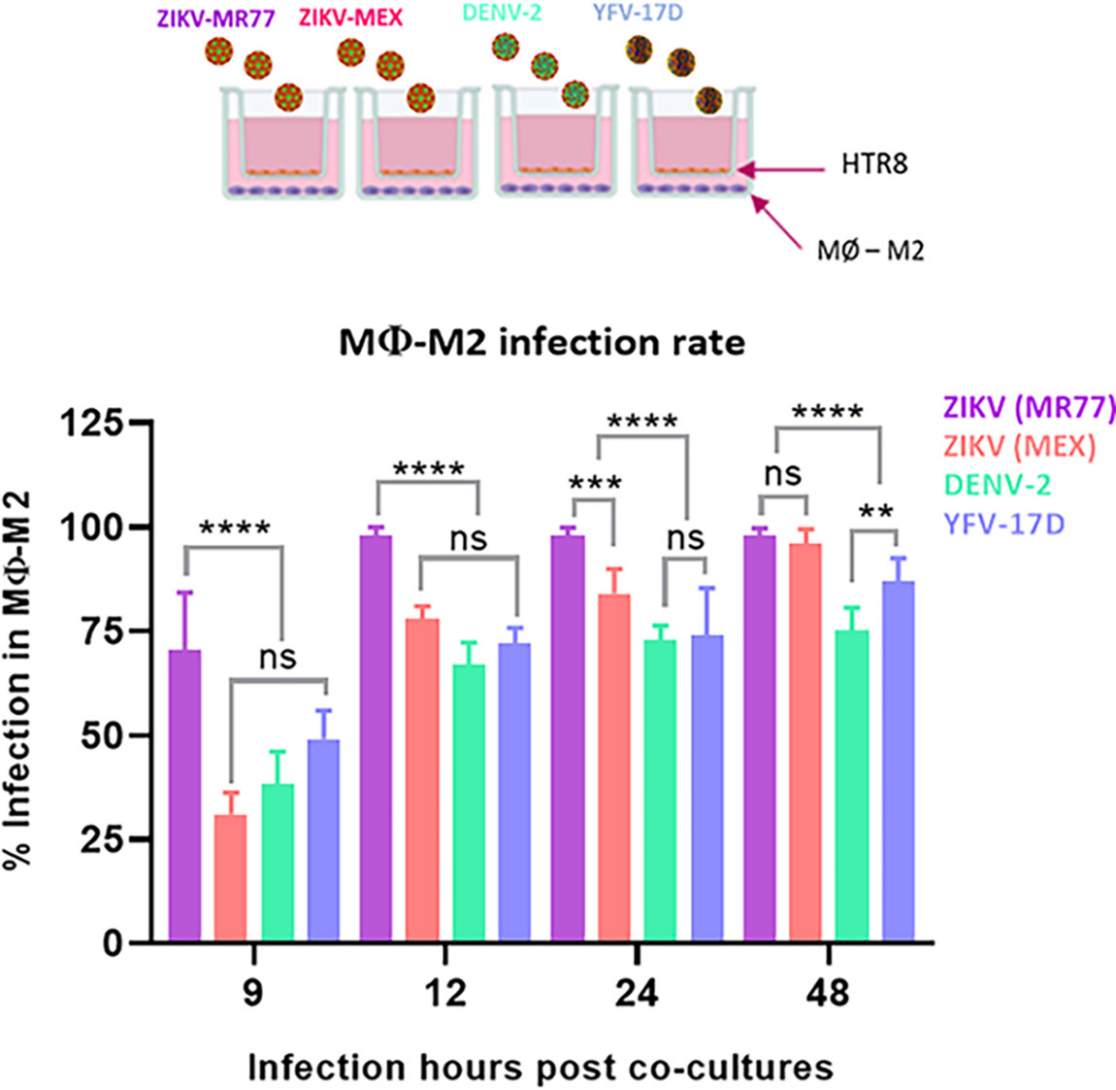
Coculture of infected HTR8 cells and differentiated M2 macrophages in a Transwell system. Confluent monolayers of HTR8 cells grown in Transwell inserts were infected with the indicated viruses (MOI = 1), and after extensive washing, the cells were cocultured with differentiated macrophages seeded in the lower chamber. The percentage of infected macrophages was determined at the indicated times by immunofluorescence using an anti-NS3 MAb as a primary antibody. *n* = 3. Bars represent means ± standard errors. ****, *P* ≤ 0.0001; ***, *P* ≤ 0.0006; **, *P* ≤ 0.0045; ns, not significant.

To further determine the growth kinetics of the different viral strains in HTR8 cells, infected HTR8 cells were cocultured with differentiated macrophages using a Transwell system. Infected HTR8 cells were seeded in the upper chamber and uninfected macrophages in the lower chamber to simulate the placental architecture. Since cultured HTR8 cells do not form tight junctions (Fig. S3), HTR8 monolayers were extensively washed before coculture to remove unbound viruses. Figure 2 shows that macrophages in the bottom chamber were first reached by ZIKV-MR77, with 100% of infected cells observed at 12 h, followed by ZIKV-MEX, with nearly 100% of infected cells observed at 48 h. In contrast, although the percentages of infected macrophages with DENV and YFV-17D increased continuously over time up to 48 h, the percentage of infected macrophages never reached 100%. These results again suggest that HTR8 cells are significantly more susceptible to ZIKV than to DENV and YFV-17D.

### mTOR pathway activation in infected HTR8 cells.

**FIG 3:**
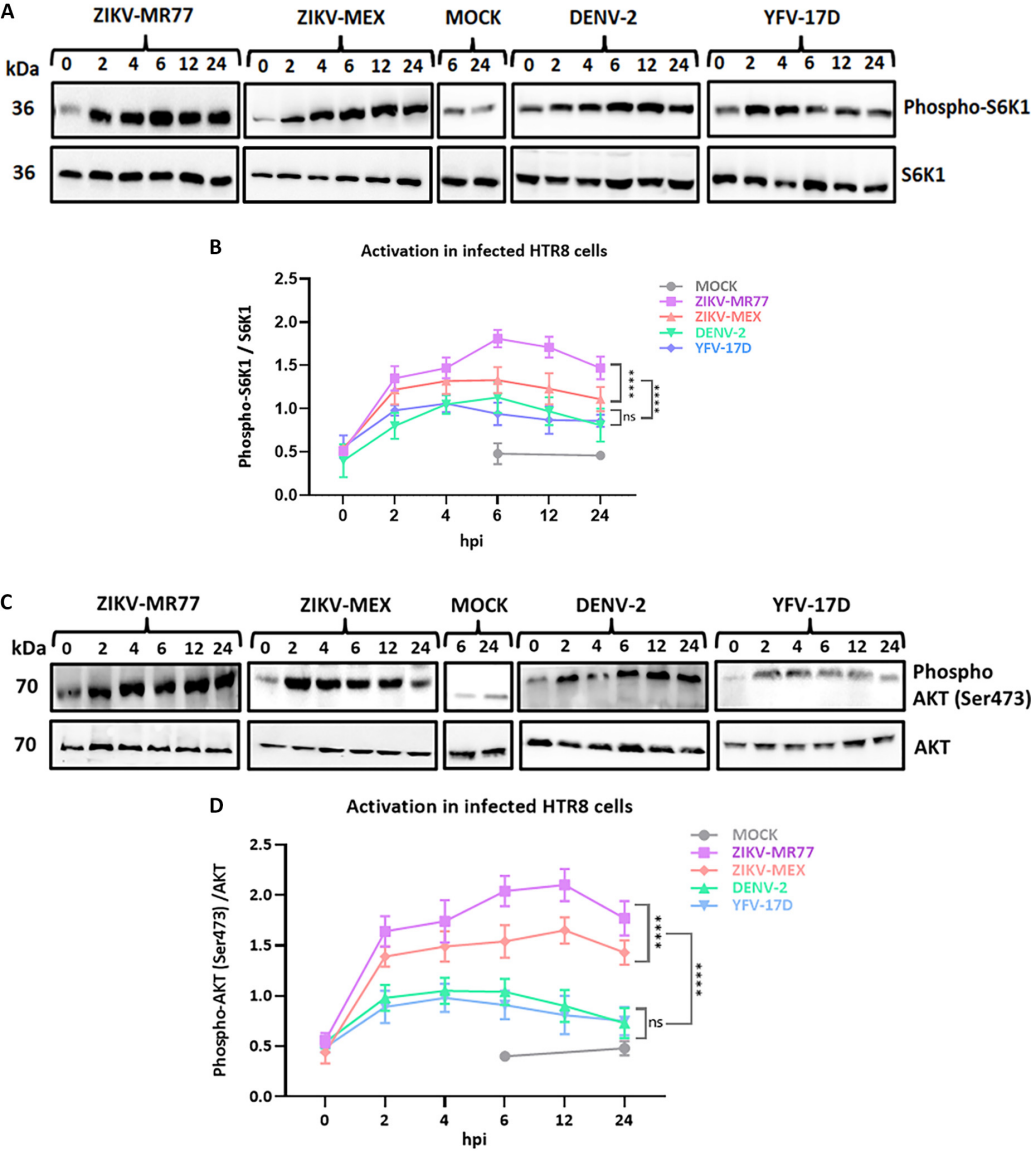
Activation of mTOR pathways in infected HTR8 cells. Confluent monolayers of HTR8 cells were infected with the indicated viruses (MOI = 3), or mock infected. Cell lysates were collected at different hours postinfection and analyzed by Western blotting, and band intensities were assessed by densitometry. Activation of the mTORC1 pathway was determined measuring the phospho-S6/S6 ratio (A and B), and the phospho-AKT/AKT ratio for the mTORC2 pathway (C and D). All experiments were carried out in triplicate. Graph lines represent means ± standard errors. ****, *P* ≤ 0.0001; ns, not significant.

The reasons that a cell is susceptible or permissive to a given viral infection are multiple. Preliminary experiments indicated that HTR8 cells do not express the classical flavivirus receptor DC-SIGN (Fig. S2). However, the mTOR signaling pathway is used by flaviviruses and other RNA viruses to promote replication and evade the antiviral response (27–33). Thus, to gain insight into the better growth capacity of ZIKV strains over DENV and YFV-17D in HTR8 cells, we evaluated the activation of mTORC1 and mTORC2 in infected cells. A faster and stronger activation of mTORC1 (Fig. 3A and B) and mTORC2 (Fig. 3C and D) in HTR8 cells infected with ZIKV strains compared with DENV and YFV-17D was observed. In ZIKV-MR77-infected cells, the activation of mTORC1 and mTORC2 peaked at 6 and 12 hpi, respectively, and doubled compared with DENV and YFV-17D. mTORC1 and mTORC2 activation in ZIKV-MEX-infected cells was also significantly higher than that induced after DENV and YFV-17D infection but less than that induced by infection with ZIKV-MR77 (Fig. 3C and D). Finally, infection with DENV and YFV-17D activated mTOR signaling to a similar extent—about 0.5-fold over background levels (mock-infected cells). The parallels observed between the growth efficiency of each of the tested strains and their capacity to activate the mTOR pathways in HTR8 cells, namely, ZIKV-MR77 > ZIK-MEX ≫ DENV and YFV-17D, suggest that mTOR pathway activation capacity and growth efficiency are related.

### Effect of rapamycin and AZD8055 treatment of infected HTR8.

**FIG 4:**
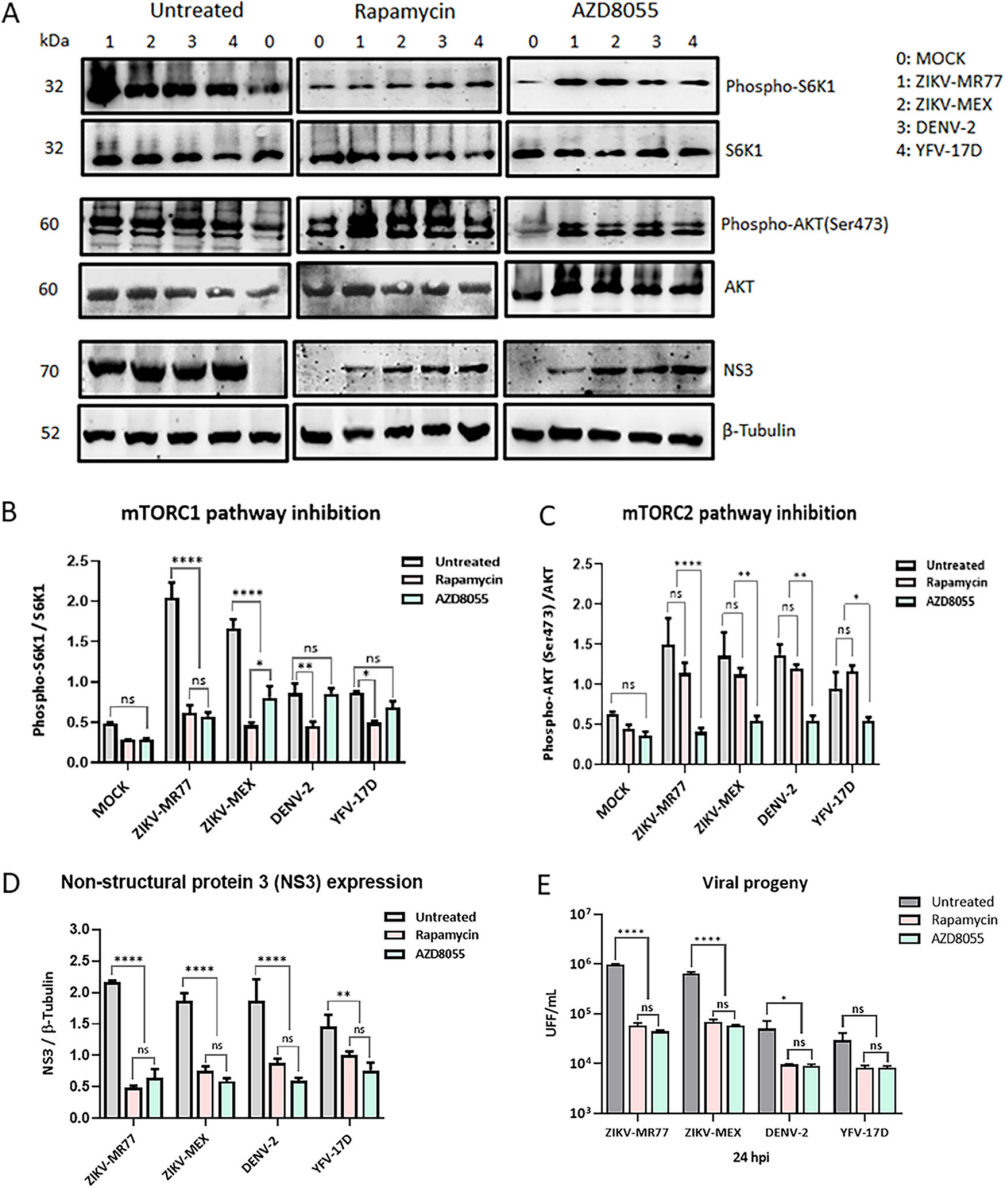
Effect of inhibition of the mTOR pathways on virus replication. HTR8 cells were pretreated with rapamycin or AZD8055 for 24 h before viral infection. Twenty-four hours postinfection, cells were lysed and analyzed by Western blotting (A), and band intensities were assessed by densitometry to assess the drug effect on mTORC1 (B) and mTORC2 (C) inhibition and NS3 expression levels (D). Supernatants were analyzed by focus assay for virus titer determinations (E). Bars show means ± standard deviations from at least 3 independent experiments. ****, *P* ≤ 0.0001; **, *P* ≤ 0.0065; *, *P* ≤ 0.0229; ns, not significant.

To test if mTOR activation may play a role in the augmented susceptibility of HTR8 cells to ZIKV infections, the dependency of the ZIKV strains’ replication on the activation of the mTOR pathways was evaluated using rapamycin and AZD8055, two allosteric inhibitors of the mTOR pathways (34, 35). Both viral NS3 protein production and virus yield were evaluated at 24 hpi. HTR8 cells remain metabolically viable 48 h after treatment, even at the highest concentrations tested: 100 nM for rapamycin and 10 μM for AZD8055 (Fig. S4). Furthermore, treatments were specific given that rapamycin inhibited mTORC1 activation, without affecting mTORC2, while AZD8055 inhibited both pathways (Fig. 4A, B, and C). Both drugs significantly reduced NS3 protein levels (Fig. 4A and D). Viral titers for all 4 strains were also reduced in rapamycin- and AZD8055-treated cells. However, while a reduction of more than 1 log in virus yield was observed for the ZIKV-MR77 and ZIKV-MEX strains (*P* ≤ 0.0001), the reduction observed for DENV was less than 1 log (*P* ≤ 0.05) and resulted in a not significant difference for YFV-17D strains. Of note, no significant differences in virus titers were observed in cells treated with rapamycin or AZD5088, indicating that it is the mTORC1 pathway that is mainly required for viral replication (Fig. 4E). The results obtained after the pharmacological inhibition of the mTOR pathways are in line with the notion that a more potent activation of the mTOR pathways is at least partially responsible for the better growth capacity of ZIKV in HTR8 cells.

### Cytokine and chemokine responses in infected HTR8 cells and M2 macrophages.

**FIG 5:**
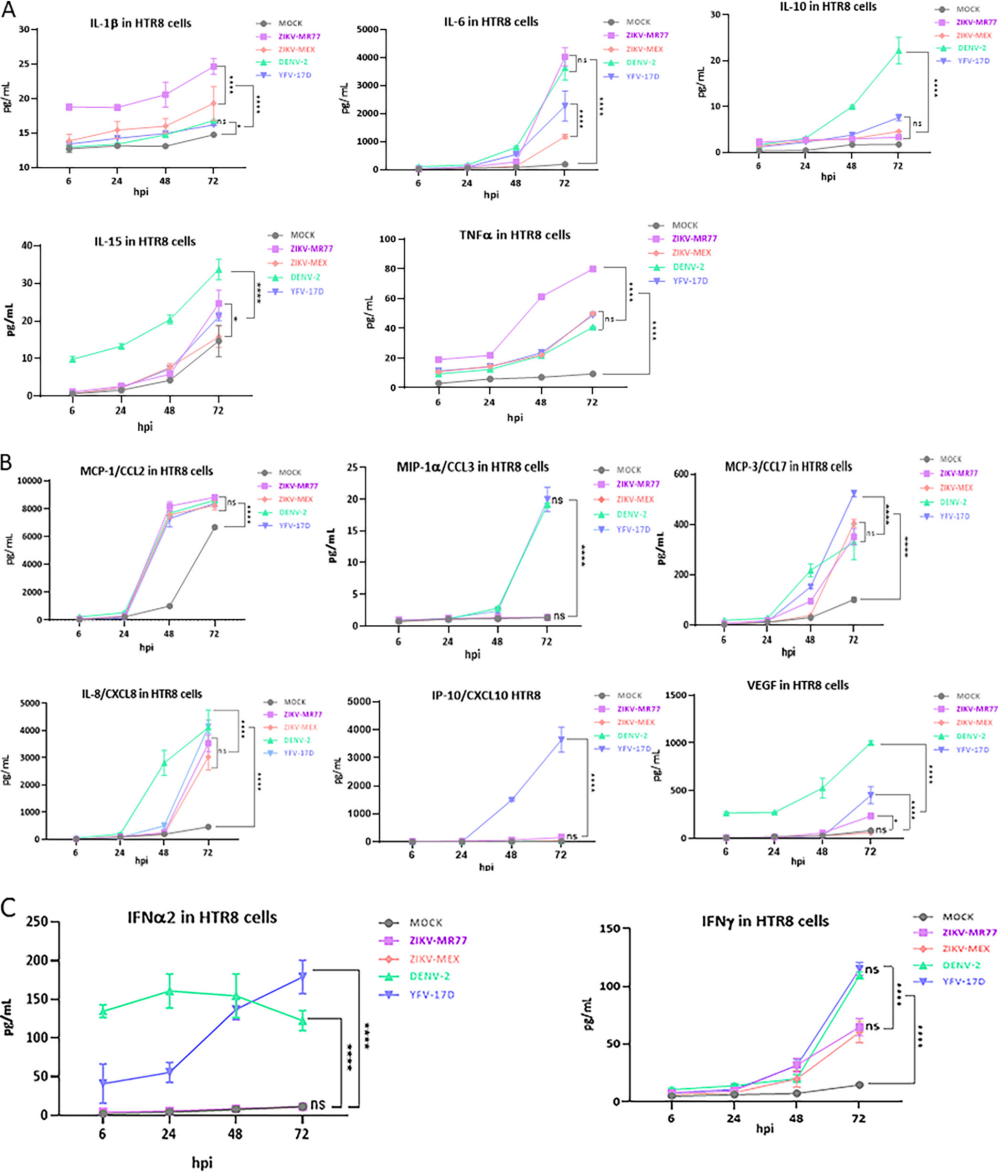
Cytokine profiles produced by infected HTR8 cells. Confluent monolayers of HTR8 cells seeded in 24-well plates were infected (MOI = 3) with the different virus strains, and supernatants were collected at the indicated times. The concentrations of secreted cytokines, chemokines, and IFN were determined by cytometric bead arrays. Graphs show mean concentrations (picograms per milliliter) ± standard deviations from at least 3 independent experiments. ****, *P* ≤ 0.0001; *, *P* ≤ 0.046; ns, not significant.

**FIG 6:**
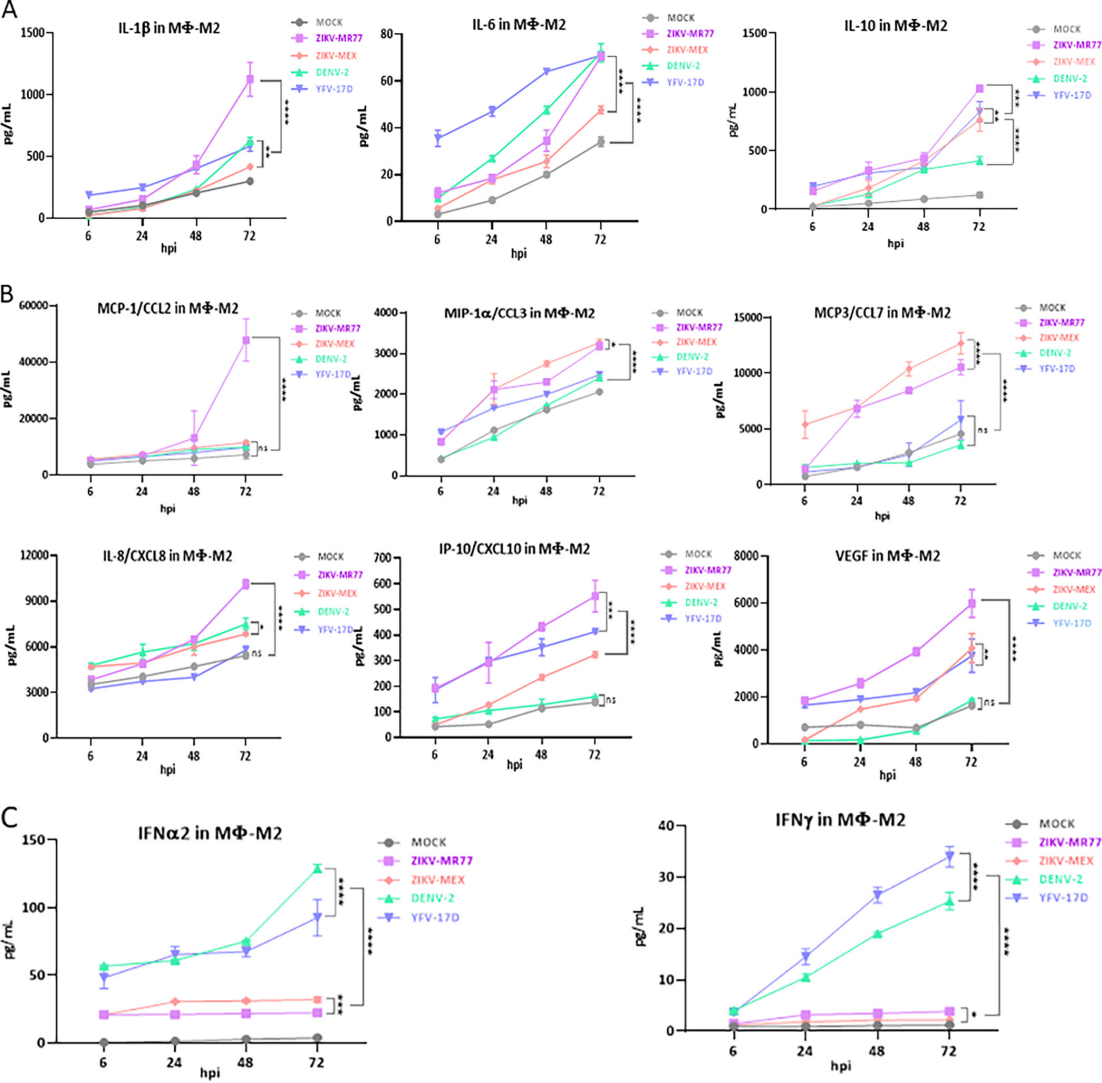
Cytokine profiles produced by infected differentiated M2 macrophages. Confluent monolayers of differentiated macrophages seeded in 24 well-plates were infected (MOI = 3) with the different virus strains, and supernatants were collected at the indicated times. The concentrations of secreted cytokines, chemokines, and IFN were determined by cytometric bead arrays. Graphs show mean concentrations (in picograms per milliliter) ± standard deviations from at least 3 independent experiments. ****, *P* ≤ 0.0001; ***, *P* ≤ 0.0008; **, *P* ≤ 0.0056; *, *P* ≤ 0.0461; ns, not significant.

The delicate balance between the anti-inflammatory and inflammatory responses is crucial for the development of the semiallogeneic fetus (15, 16). All tested cytokines were induced after infection by the ZIKV-MR77, ZIKV-MEX, DENV, and YFV-17D strains in both HTR8 cells and M2-Mϕs. The induction was more evident at later time points (24 to 72 hpi) and more robust in M2-Mϕs (Fig. 5 and 6; see Tables S1 and S2 in the supplemental material). The cytokine profile of HTR8 cells infected with ZIKV strains was characterized by high levels of interleukin-1β (IL-1β), IL-6, and tumor necrosis factor alpha (TNF-α) and low levels of IL-10, CCL-3, IP-10, vascular endothelial growth factor (VEGF), IFN-2α and IFN-γ (Fig. 5A, B, and C). Differences between ZIKV strains and DENV and YFV-17D were particularly evident for CCL-3, IP-10, IFN-2α, and IFN-γ, suggesting that in HTR8 cells, the infection with ZIKV inhibits the production of chemoattractant and recruiting chemokines as well as IFNs.

Interestingly, except for CCL-7, the differences in cytokine expression triggered by the ZIKV strains and DENV and YFV-17D strains were less evident in M2-Mϕs. Nonetheless, ZIKV-MR77 generated a higher inflammatory and chemotactic response, with superior values of IL-1β, IL-6, TNF-α, CCL-2, CCL-3, CCL-7, CXCL8, IP-10, and VEGF, than the rest of the strains (Fig. 6A and B). The poor IFN response induced by ZIKV strains was also observed in M2-Mϕs (Fig. 6C).

## DISCUSSION

ZIKV is a TORCH pathogen with high teratogenic potential (5); in contrast, other related flaviviruses, such as DENV and YFV, have not been associated with teratogenic fetal damage (2, 10, 13). How ZIKV crosses the blood-placenta barrier need to be deciphered, given the social and public health impact generated by CZS. Parallel infections using African and Asian lineage ZIKV strains, along with DENV and YFV-17D, in HTR8 cells suggest that these cells are significantly more susceptible to ZIKV than to DENV or YFV-17D. Previous results suggested that HTR8 cells are equally susceptible to ZIKV, DENV, and YFV (24). However, in our hands both ZIKV strains, particularly the African strain, showed significantly higher infection and replication capacity in HTR8 cells than DENV and YFV-17D. Differences in the methodologies used in this work to measure virus growth, such as classical one-step growth curves and cocultures in Transwells versus titer determinations at 1, 4, and 6 days postinfection, may account for these differing results. On the other hand, M2-Mϕs were highly permissive to all four strains, and the marked differences observed in HTR8 cells were reduced, most likely reflecting the high susceptibility of cells of the monocytic lineages to all mosquito-borne flaviviruses (36, 37). Note that HBCs have been implicated in the vertical transmission of ZIKV (18, 19). The results obtained in both cell lines suggest a role for CTB cells in gating the pass of ZIKV, but not DENV or YFV, to the placental stroma and that once inside the stroma, ZIKV is amplified by HBCs, favoring virus spread to the fetal circulation (18, 19).

Congenital malformations associated with ZIKV have been attributed only to the Asian lineage (3, 4). However, our findings agree with previous reports using human placental trophoblasts, animal cell lines, mosquitoes, mouse models, and, more recently pregnant higher primate models, which all evidenced the higher capacity for transmission, replication, and virulence of the African over the Asian lineage (38–40). Thus, our data add to the accumulating evidence that African lineage ZIKV strains may be more virulent and more pathogenic to pregnancy than Asian lineage strains, generating early miscarriages and preventing advanced fetal development (41).

Cell permissiveness to a viral infection is the result of multiple factors. The mTOR machinery is a cell signaling pathway related to the regulation of various processes such as growth, proliferation, survival, protein synthesis, ribosome and lipid biogenesis, autophagy, and migration (42, 43). This pathway has been reported to be essential during replication and induction of antiviral immune responses by various RNA viruses, including ZIKV, DENV, and YFV (27–33, 44, 45). Our results suggest that in HTR8 cells, ZIKV, especially the African strains, activates mTOR signaling far more effectively than DENV and YFV-17D. Inhibition by chemical means of mTOR signaling resulted in decreased NS3 viral protein expression and lower viral titers under all infection conditions. The stronger inhibition in the replication of ZIKV after rapamycin and AZD8055 treatment demonstrated a higher reliance of the ZIKV strains on the activation of mTOR and specifically the activation of mTORC1.

Our results agree with and expand those from previous works reporting the activation and the dependency on the activation of the AKT/mTORC pathways of ZIKV infections, using liver, neuroblastoma, neural progenitor, and embryonic kidney human cell lines (2, 28, 30, 31). In all of those relevant cell models, inhibition of the mTOR or phosphatidylinositol 3-kinase (PI3K)/AKT pathways adversely affects ZIKV replication. mTORC1 activation in ZIKV-infected cells has been associated with negative regulation of autophagy (27, 28, 30, 46) and enhanced NS5 polymerase activity to facilitate viral replication (31). In addition, general processes such as viral protein synthesis (31, 32, 44, 45) and endoplasmic reticulum (ER) membrane remodeling (47) are also presumably favored by mTOR modulation. In agreement, mTOR signaling inhibition in BHK-21 cells reduced YFV infection (27).

However, Liang et al. (45) observed no activation, but inhibition of the AKT/mTOR pathway in ZIKV (African lineage)-infected human fetal neural stem cells, leading to defective neurogenesis and aberrant autophagy, although the effect on virus replication was not directly evaluated. Conflicting results regarding mTOR activation have also been observed for cells infected with DENV. Inhibition of mTOR signaling in DENV-infected HepG2 cells (47), BHK-21 cells and mouse neuroblasts (48), human umbilical vein endothelial cells (HUVECs) (31), and megakaryocytes (49) accelerated the infection. These differences may be regulated the fact that contrary to ZIKV infection, DENV infection seems to be favored by autophagy (31, 47).

Many works infer that the innate immune response contributes to the vertical transmission of the Zika virus and the development of SCZ (50, 51). The results in HTR8 cells indicate that ZIKV infection negatively modulates the chemotactic and interferon responses in CTBs. Similar results using the same cell line were reported by Luo et al. (24): higher levels of IL-6 and TNF-α and lower levels of CCL3 and IFN-α/β secretion were observed for ZIKV infection than for DENV-4 and YFV-17D. Additionally, in another study conducted in human brain astrocytes, the high rate of viral replication of both African and Asian strains of ZIKV was directly related to a limited response of proinflammatory and chemotactic cytokines (IL-6, IL-8, IL-12, CCL2, CCL5, and IP-10) (52).

The innate response was more robust in macrophages than in HTR8 cells, and the differences between ZIKV strains and DENV and YFV-17D were less noticeable. Infection with both ZIKV lineages was characterized by a more proinflammatory and chemotactic profile, mediated by high levels of IL-1β, IL-6, TNF-α, CCL-2, CCL-3, CCL-7, CXCL8, IP-10, and VEGF. Yet, the IFN-α/γ response was equally reduced in macrophages infected with ZIKV, compared to the DENV and YFV-17D strains. The ability of ZIKV to negatively modulate the interferon response has been reported (53–55). Proposed mechanisms to explain the low IFN induced by ZIKV infections include proteasomal degradation of the STAT2 activator (54) and modulation of the biogenesis of peroxisomes, the signaling platforms during the IFN response (55). Whether these mechanisms operate in ZIKV-infected HTR8 cells needs to be investigated. The cytokine profiles observed in this work suggest that the establishment of ZIKV infection in the placental stroma is favored by the limited inflammatory, chemotactic response and the low levels of IFN-α/γ induced in CTBs, thus positively modulating their permissibility to ZIKV replication. Likewise, the low IFN response in macrophage cells may be decisive in the propagation of ZIKV to fetal circulation.

A major limitation of this study is the sole use of immortalized cell lines in culture. HTR8 cells were derived from extravillous trophoblasts and developed as a placental study model to overcome the many experimental and ethical difficulties encountered when working directly with placental tissues, especially from the first trimester. Indeed, attempts to isolate HBCs from term placentas were unsuccessful in our hands. HTR8 cells are commonly used as a trophoblast model in placenta-related studies; however, their transcription signature indicated that while a number of genes are expressed in common with invasive primary trophoblast cells, others are not (56). Similarly, albeit HBCs are induced by macrophage colony-stimulating factor (M-CSF) and express several M2 markers, such as CD163, CD209, and CD14, also expressed by the M-CSF-differentiated U937 cells used in this study, they seem not to be typical M2 macrophages (26). Thus, the results hereby presented, especially those obtained with HTR8 cells, will require verification in a more suitable placental model, such as primary cell lines or *ex vivo* placental cultures.

In summary, these results suggest that the CTB constitutes a physical barrier that helps to contain DENV and YFV infection but is successfully surpassed by ZIKV. Once inside the placental stroma, ZIKV will target HBCs, where the infection is amplified, subsequently spreading to the fetal circulation. The capacity of the ZIKV to cross the CTB barrier seems to be the result of a sum of factors, including greater infection and replication efficiency, better capacity to activate the mTOR machinery, and a limited inflammatory and chemotactic response with low IFN activity.

## MATERIALS AND METHODS

### Cell lines.

HTR8-SVneo cells, derived from human, first trimester of gestation placental trophoblasts (ATCC; CRL-3721), and U937-DC-SIGN cells, from human prohistiocytic lymphoma (ATCC; CRL-3253), were grown in RPMI 1640 medium (Gibco; 11875-093); Vero-E6 cells (ATCC; CRL-1586) and mosquito C6/36 cells, derived from Aedes albopictus (ATCC; CRL-1660), were grown in Eagle’s minimum essential medium (EMEM). All media were supplemented with 10% fetal bovine serum (FBS) and 100 U/mL penicillin-streptomycin. Mammalian cells were grown at 37°C and mosquito cells at 28°C with 5% CO_2._

### Virus strains.

The Zika virus strain Uganda (ZIKV-MR77), African genotype, was donated by Susana López, (IBT-UNAM, Cuernavaca, Mexico). The Zika virus Mexican strain (ZIKV-MEX), Asian genotype, and the dengue virus serotype 2 strain New Guinea were provided by Mauricio Vázquez (InDRE, Mexico City). The YFV-17D vaccine strain was donated by Juan Salas-Benito (IPN, Mexico City). Viral strains were all propagated in C6/36 cells and titrated by focus assay in Vero-E6 cells.

### Monocyte differentiation to M2 macrophages.

U937-DC-SIGN cells were treated for 12 days with 160 nM phorbol 12-myristate-13-acetate (PMA) (Sigma-Aldrich; P1585) and 30 ng/mL macrophage-colony-stimulating factor (M-CSF) (Sigma-Aldrich: M6518), with the medium replaced every third day (25, 57). The percentage of differentiated monocytes was determined by flow cytometry and fluorescence microcopy, using the following markers, all present in M2 placental macrophages (M2-Mϕs): CD163, CD209 (DC-SIGN), and CD14 (26, 58).

### One-step growth curves.

Confluent monolayers of HTR8 cells and differentiated monocytes were seeded in 96-well plates and infected in triplicate with ZIKV-MR77, ZIKV-MEX, DENV, and YFV-17D strains using a multiplicity of infection (MOI) of 3. Virus binding was carried out for 40 min at 4°C to synchronize the infections. Afterward, cells were washed 3 times with cold phosphate-buffered saline (PBS), maintenance medium was added, the cells were switched to 37°C, and infections were allowed to proceed for 0, 6, 18, 24, 48, 72, and 96 h. At these times, supernatants were collected, and stored at −80°C until titration (19). Briefly, Vero cells seeded in 96-well plates were infected with 10-fold serial dilutions of the supernatants. At 48 hpi, cells were fixed with cold methanol, washed with PBS, and stained using anti-E monoclonal antibody (MAb) (4G2) as a primary antibody, and an anti-mouse IgG-peroxidase conjugate (Jackson Immuno-Research; 15-035-003) as a secondary antibody. Infected cells were visualized using 3,3′-diaminobenzidine (DAB) (Vector Laboratories; SK-4100). Virus titers were expressed as FFU per milliliter (46).

In addition, the HTR8 and M2-Mϕ monolayers from the one-step replication curves were used to determine the percentage of infected cells at 24, 48, 72, and 96 hpi, by immunofluorescence. Briefly, monolayers were fixed in 4% paraformaldehyde for 10 min and permeabilized with 0.1% Triton X-100 for 10 min at room temperature. Cells were stained using a cross-reactive anti-NS3 MAb (1ED8) as a primary antibody and an anti-mouse Alexa 488-conjugated donkey antibody, preadsorbed (Abcam; ab150064), as a secondary antibody. Nuclei were counterstained with DAPI (4′,6-diamidino-2-phenylindole), and cells were analyzed under a Nikon inverted microscope (Eclipse Ti-U). The virus yield/cell was calculated by obtaining the ratio between viral progeny and the percentage of infected cells, assuming 10 × 10^5^ cells/well, after confluence.

### Cocultures of HTR8 cells and differentiated macrophages in Transwells.

Infected HTR8 cells and U937 monocytes differentiated to M2-Mϕs, were cocultured using a Transwell system (12-well plate, 0.4 μm) (catalog no. 3460; Corning, Kennebunk, ME). Inserts containing confluent monolayers of HTR8 cells were infected with ZIKV-MR77, ZIKV-MEX, DENV, or YFV-17D at an MOI of 1 for 1 h at 4°C to allow absorption, followed by 2 h at 37°C. After removal of the inoculum, HTR8 monolayers were washed three times with PBS, and the inserts were placed on plates seeded in the lower chamber, with noninfected M2-Mϕs differentiated for 12 days. The cocultures were incubated at 37°C, and the percentage of infected M2-Mϕs was determined after 9, 12, 24, and 48 h by immunofluorescence, as described above.

### mTOR activation in infected HTR8 cells.

Activation of the mTORC1 and mTORC2 pathways was evaluated by Western blotting, analyzing the status of phospho-S6r for mTORC1 and phosphor-AKT-Ser473 for mTORC2. Infected drug-treated or control cells were lysed in radioimmunoprecipitation assay (RIPA) lysis buffer (5 M NaCl, 0.5 M EDTA [pH 8], 10% sodium deoxycholate, 10% SDS, 10% Triton X-100) with protease inhibitor cocktail (Sigma-Aldrich; P8340). The protein concentration in the extracts was quantified using the Bradford protein assay (Bio-Rad; 500-0006). Depending on the experiment, 15 or 50 μg of protein was processed for 10% SDS-PAGE. Proteins were transferred to nitrocellulose membranes (0.45 μm pore) (Bio-Rad) following standard protocols. The following primary antibodies were used: a rabbit MAb to S6 ribosomal protein (Cell Signaling; 2217) and a rabbit MAb to phospho-S6 ribosomal protein (Ser235/236) (Cell Signaling; 4858) for the mTORC1 pathway and rabbit MAbs to AKT1 (Cell Signaling; 2938) and phospho-AKT (Ser473) (Cell Signaling; 4060) proteins for the mTORC2 pathway. An anti-rabbit antibody conjugated to horseradish peroxidase (HRP) (GeneTex; GTX-26721) was used as a secondary antibody. All antibodies were diluted in 5% skim milk powder with PBS and 0.1% Tween 20. The HRP signal was detected using the SuperSignal West Femto kit (Thermo Fisher Scientific; 34096). Phosphorylated fractions were normalized with the same nonphosphorylated protein fraction. Digital images were captured with the ImageQuant LAS 4000 system (GE Healthcare) and analyzed with ImageJ software.

### Inhibition of mTOR pathways in infected HTR8 cells.

Rapamycin (Tocris-Bioscience; 53123-88-9) was used to inhibit the mTORC1 pathway, and the ATP competitor AZD8055 (AstraZeneca; 2525) was used for inhibition of the mTORC1 and mTORC2 pathways. Cells were treated with 100 nM rapamycin or 10 μM AZD8055, diluted in dimethyl sulfoxide (Sigma-Aldrich: D-2650), for 24 h before infection. These concentrations proved to be nontoxic to HTR8 cells (see Fig. S4 in the supplemental material). Afterward, cells were infected at an MOI of 3 with each viral strain; supernatants were collected (24 hpi) for virus yield determination by focus-forming assay. In addition, viral protein expression was detected using the anti-NS3 mouse MAb 1ED8 and β-tubulin (GeneTex; GTX-101279) as load control. An anti-mouse antibody conjugated to HRP (Jackson Immuno-Research; 15-035-003) was used as a secondary antibody.

### Cytokine and chemokine responses in infected cells.

HTR8 cells and M2-Mϕs were either mock infected or infected at an MOI of 3 with ZIKV-MR77, ZIKV-MEX, DENV-2, and YFV-17D. Cell supernatants were collected at 6, 24, 48, and 72 hpi and analyzed in triplicate for the presence of the cytokines and chemokines IL-1β, IL-6, IL-8/CXCL8, IL-10, IL-15, TNF-α, MCP-1/CCL-2, MIP-1α/CCL-3, MCP3/CCL-7, IP-10/CXCL10, VEGF, IFN-2α, and IFN-*y* using a commercial multiplex system (Bio-Plex multiplex immunoassay system) (HCYTOMAG 13K; Merck). Samples were processed following the supplier’s instructions, and cytokine concentrations were determined in a Luminex X-200. Results were plotted as line charts using GraphPad Prism software v.6.01.

### Statistical analysis.

Values obtained from all experiments were expressed as means ± standard errors (one-way analysis of variance [ANOVA]) from at least three independent experiments. Statistical analyses and graphs were performed with GraphPad Prism software v.6.01.
