# Supplementary material for: Comparative Infections of Zika, Dengue, and Yellow Fever Viruses in Human Cytotrophoblast-Derived Cells Suggest a Gating Role for the Cytotrophoblast in Zika Virus Placental Invasion
Source: Microbiol Spectr. 2023 May 25;11(3):e00630-23. doi: 10.1128/spectrum.00630-23 (PMC10269719; doi:10.1128/spectrum.00630-23)
Supplement: Supplemental file 1 — Supplemental material. Download spectrum.00630-23-s0001.docx, DOCX file, 7.5 MB [file spectrum.00630-23-s0001.docx]

**
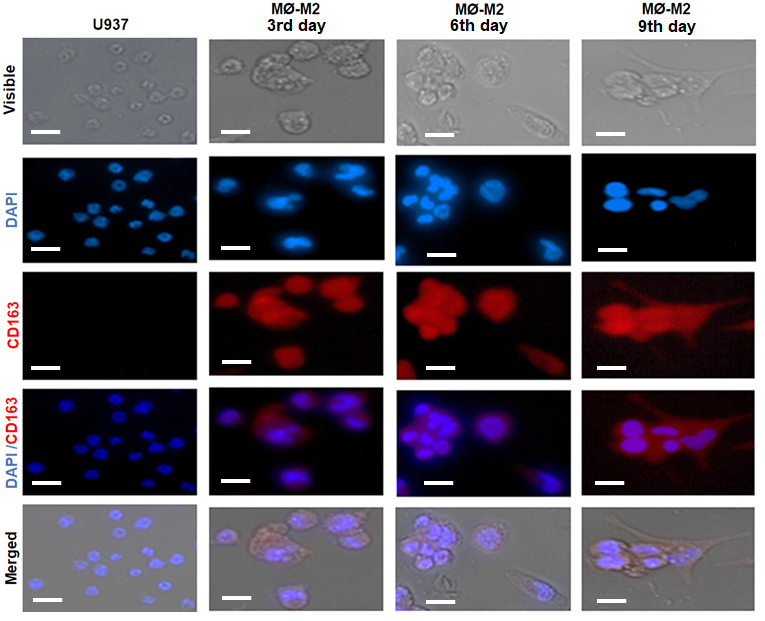
Supplemental Figures**

**Supplemental figure 1. Monocyte differentiation determined by epifluorescence microscopy.** U937-DC-SIGN cells were grown in 24-well plates containing glass coverslips and treated with PMA and M-CSF at the concentrations described above during 3, 6 and 9 days; untreated U937-DC-SIGN were included as controls. At the indicated times, cells were washed once with PBS, fixed in paraformaldehyde 4% for 10 min, and permeabilized with 0.1% Triton X-100 for 10 min at room temperature. Cells were stained using a Mab CD-163 (Abcam®: ab182422), as primary antibody, and an anti-rabbit Alexa-594 donkey pre-adsorbed (Abcam®: ab150064) as secondary antibody. Coverslips were mounted in Fluoroshield™ with DAPI (Sigma-Aldrich®: F6057) and analyzed under an inverted Nikon microscope (model Eclipse Ti) (Scale bar 20 µm).

**
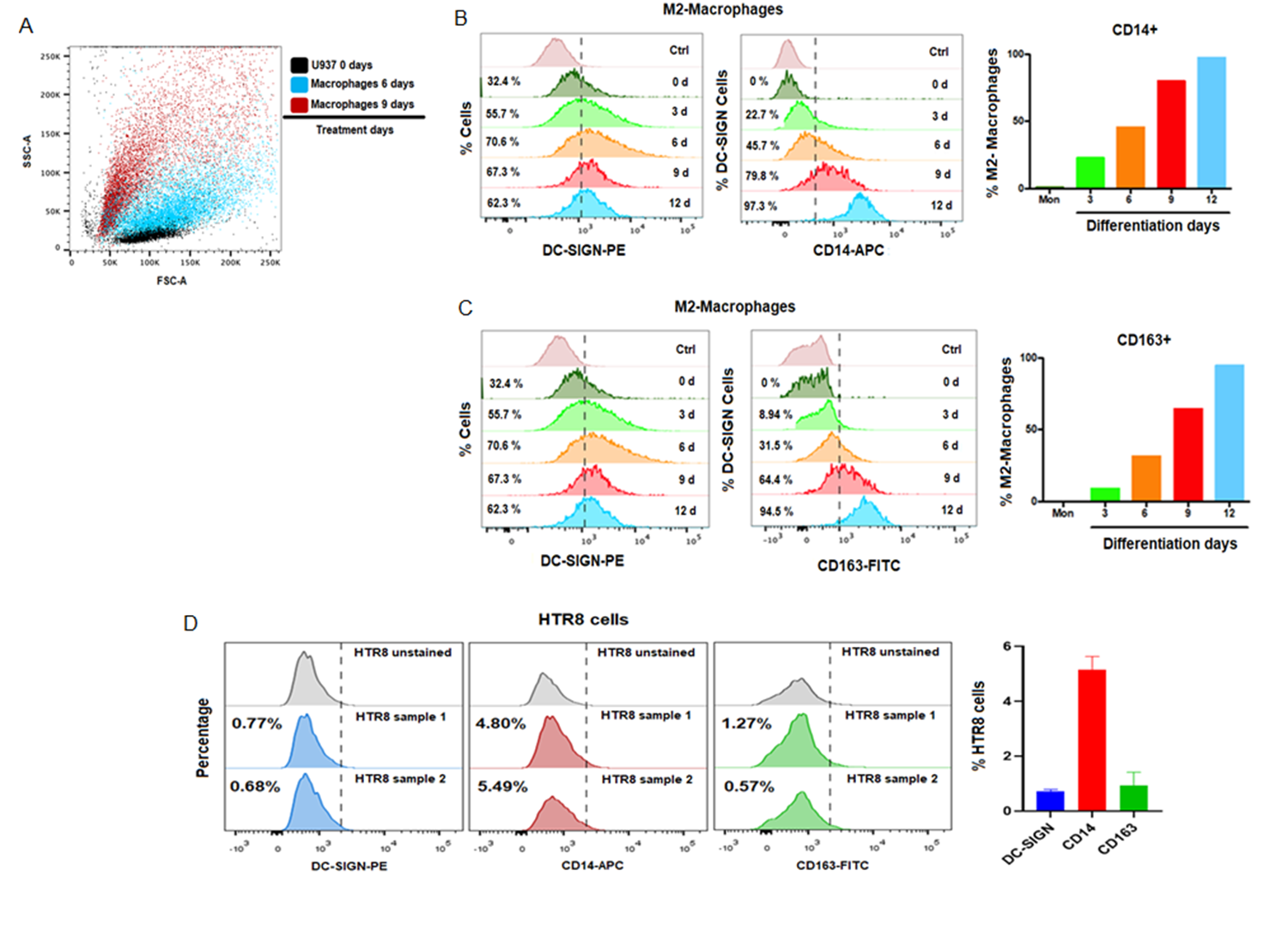
**

**Supplemental figure 2. Expression of DC-SIGN, CD14 and CD163 receptors in MØ-M2 and HTR8 cells.** Monocytes U937-DC-SIGN differentiated into M2-MØ and HTR8 cells were fixed in suspension with paraformaldehyde 4% for 5 min at room temperature and washed twice in phosphate buffer saline (PBS). Fixed cells were incubated with PBS and 5% FBS for 10 min in ice, to block antibody unspecific binding. Finally, cells were stained for three characteristic markers of macrophages M2: CD14, using an APC anti-CD14 mouse monoclonal (Abcam®: 60901); CD163, using a FITC anti-CD163 mouse monoclonal (BD Bioscience®: 563697), and CD209 (DC-SIGN), using a PE anti-DC-SIGN mouse monoclonal (Abcam®: 136333). All antibodies were diluted 1:100 in a final volume of 50 µL. For all conditions, cells were incubated with the antibody mixtures in ice for 1 hour, in the darkness and washed twice in PBS. Appropriate unstained controls were included for each antibody. Stained cells were analyzed in a flow cytometer BD-LSR II Fortessa, and at least 10,000 events were recorded. Graphics made with the software FlowJo v10.6.1. **A.** Analysis of SSC-A (cytoplasmic complexity) versus FSC-A (size). **B.** CD14 expression from in U937-DC-SIGN positive cells at 0, 3, 6, 9 and 12 days of differentiation. **C.** CD163 expression from in U937-DC-SIGN positive cells at 0, 3, 6, 9 and 12 days of differentiation. **D**. Expression of DC-SIGN, CD14 and CD163 in HTR8 cells.


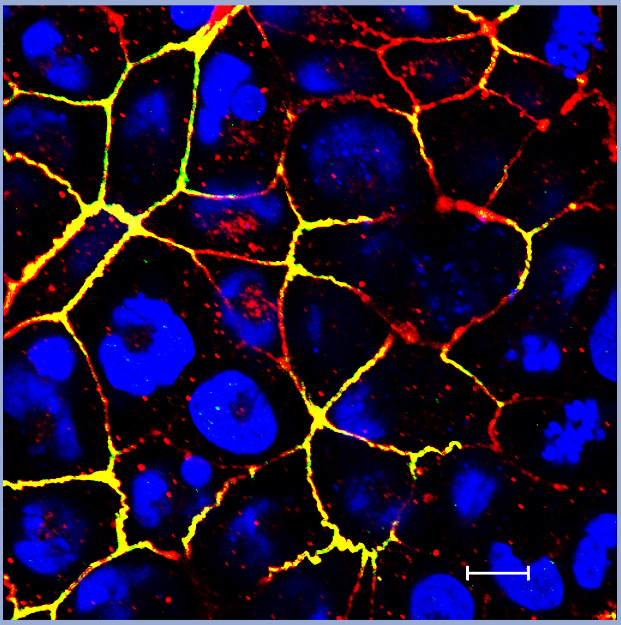

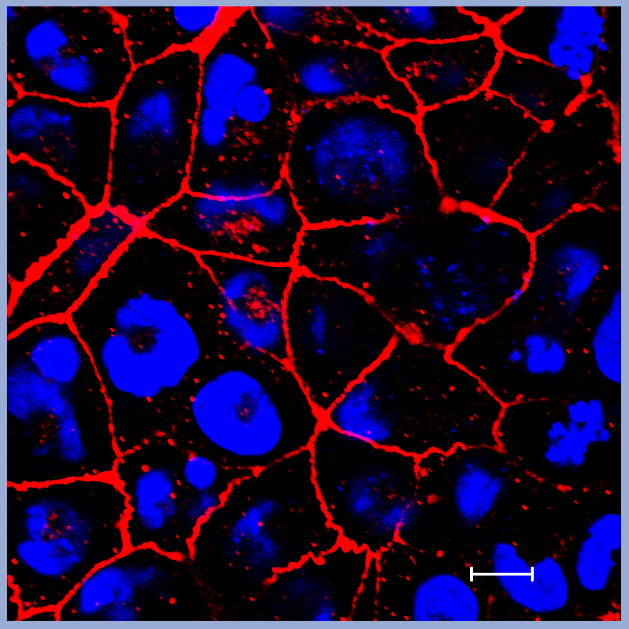

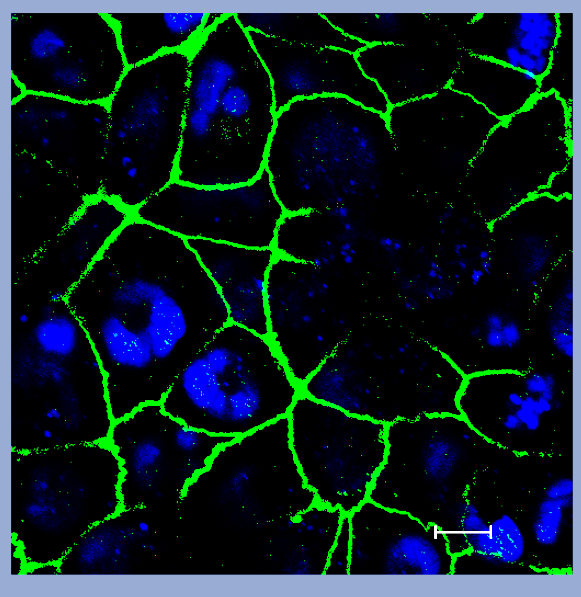

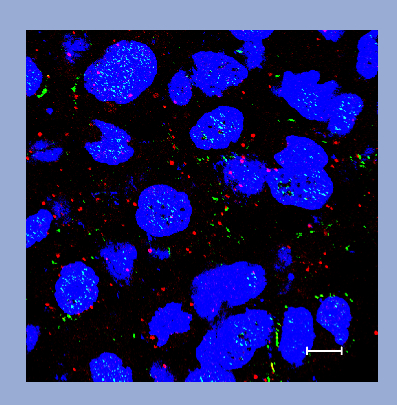

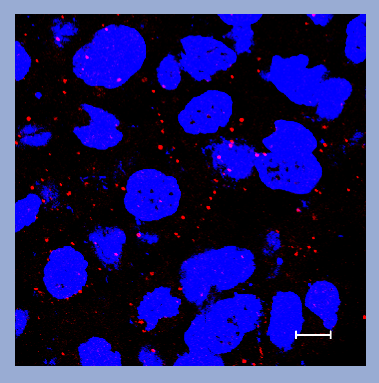

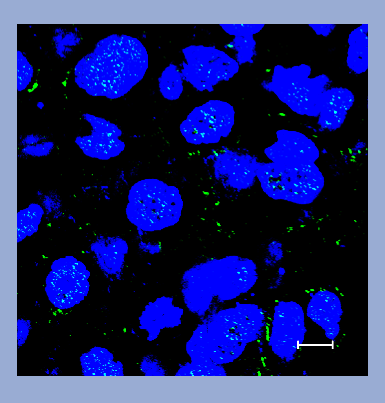


**ZO-1**

**Occludin**

**Merged**

**Supplemental figure 3. Expression of tight junction proteins.** HTR8 cells (lower panels) and Caco-2 cells (upper panels) used as positive controls, were cultured until confluency on glass cover slips, inside 24 well-plates. The expression of the tight junction proteins occludin and ZO-1 was evaluated using commercial primary antibodies (anti-occludin antibody produced in mouse, Invitrogen: A11006; anti-ZO-1 antibody produced in rabbit; Invitrogen:402200) and followed by Alexa conjugated secondary antibodies. Nuclei were counterstained with DAPI. Cells were examined in a confocal Zeiss LSM 700 microscope. Bar = 10 µM.

**Supplemental figure 4. Cell viability assay in HTR8 cells treated with rapamycin and AZD8055.** Cells were seeded in 96-well plates, then treated with rapamycin and AZD8055 at the indicated concentrations, including non-treatment and DMSO, control conditions. Plates were incubated with the drugs for 48 hours. Cell viability was determined using Cell Titer 96 AQueous nonradioactive cell proliferation assay (MTS assay) (Promega®: G3580) used according to the manufacturer’s procedures. n=3.

**Supplemental table. 1: Profile of cytokines, interferons and chemokines secreted by HTR8 cells infected with the different flaviviruses.** The supernatants of each condition were collected at different times (6, 24, 48 and 72 hpi). Each value represents the mean concentrations (pg/mL) + standard deviation of three independent experiments (****p, < 0.0001; *p, < 0.046; ns, not significant).

| **Condition** | **hpi** | **Cytokines (pg/mL)** | | | | | **Interferons (pg/mL)** | |
| --- | --- | --- | --- | --- | --- | --- | --- | --- |
|  |  | **IL-1β** | **IL-6** | **IL-10** | **IL-15** | **TNFα** | **INF-2α** | **INF-*y*** |
| ***MOCK*** | *6*  *24*  *48*  *72* | 13 + 0.5  13 + 0.1  13 + 0.1  15 + 0.1 | 14 + 0.2  54 + 2  76 + 3  192 + 1 | 0.4 + 0.1  0.5 + 0.1  2 + 0.1  2 + 0.1 | 0.6 + 0.1  2 + 0.1  4 + 0.1  15 + 4 | 3 + 0.3  6 + 0.3  7 + 0.1  9 + 0.1 | 2 + 0.3  4 + 2  8 + 2  11 + 1 | 5 + 1  6 + 0.2  7 + 1  15 + 1 |
| ***ZIKV-MR77*** | *6*  *24*  *48*  *72* | 19 + 0.5  19 + 0.5  21 + 2  25 + 1 | 15 + 1  82 + 1  275 + 22  4025 + 330 | 2 + 0.7  3 + 0.2  3 + 0.1  3 + 0.1 | 1 + 0.1  3 + 0.5  6 + 0.6  25 + 4 | 19 + 0.2  22 + 0.3  61 + 0.1  80 + 0.4 | 4.4 + 2  6 + 3  9 + 3  11 + 1 | 7. + 1  10 + 3  32 + 6  65 + 8 |
| ***ZIKV-MEX*** | *6*  *24*  *48*  *72* | 14 + 1  16 + 1  16 + 1  19 + 3 | 11 + 1  45 + 1  117 + 1  1177 + 80 | 1 + 0.2  3 + 0.1  3 + 0.1  5 + 0.1 | 0.6 + 0.1  2 + 0.2  8 + 1  16 + 3 | 11 + 0.5  14 + 0.1  22 + 0.2  50 + 0.2 | 4 + 2  6 + 1  9 + 3  11 + 3 | 6 + 1  8 + 2  20 + 7  60 + 9 |
| ***DENV-2*** | *6*  *24*  *48*  *72* | 13 + 0.2  13 + 0.2  15 + 0.2  17 + 0.2 | 105 + 7  165 + 12  793 + 38  3631 + 431 | 2 + 0.7  3 + 0.1  10 + 0.2  22 + 3 | 10 + 1  13 + 1  20 + 1  34 + 3 | 9 + 0.1  12 + 0.1  22 + 0.3  41 + 0.6 | 135 + 8  161 + 22  155 + 28  123 + 13 | 10 + 1  14 + 2  20 + 3  109 + 3 |
| ***YFV-17D*** | *6*  *24*  *48*  *72* | 13 + 0.2  14 + 0.2  15 + 0.2  16 + 0.2 | 30 + 1  94 + 4  540 + 32  2268 + 534 | 1 + 0.5  2 + 0.1  4 + 0.5  8 + 0.7 | 0.7 + 0.2  2 + 1  7 + 1  21 + 1 | 11 + 0.4  14 + 0.1  24 + 0.3  49 + 0.1 | 41 + 25  56 + 13  137 + 13  179 + 22 | 8 + 2  11 + 4  31 + 5  115 + 6 |

Supplemental table 1, cont.

| **Condition** | **hpi** | **Chemokines (pg/mL)** | | | | | |
| --- | --- | --- | --- | --- | --- | --- | --- |
|  |  | **MCP1 / CCL2** | **MIP1α / CCL3** | **MCP3 /CCL7** | **IL-8 / CXCL8** | **IP-10 / CXCL10** | **VEGF** |
| ***MOCK*** | 6  24  48  72 | 39 + 3  242 + 17  1014 + 102  6677 + 104 | 0.7 + 0.2  1.1 + 0.1  1.2 + 0.2  1.3 + 0.2 | 1.7 + 0.5  12 + 2  30 + 1  101 + 9 | 15 + 2  84 + 6  193 + 21  466 + 52 | 2 + 0.2  6 + 0.7  14 + 3  21 + 3 | 6 + 0.2  19 + 3  29 + 0.1  81.4 + 0.1 |
| ***ZIKV-MR77*** | 6  24  48  72 | 87 + 10  299 + 7  8185 + 329  8825 + 112 | 1 + 0.3  1.2 + 0.4  1.3 + 0.4  1.4 + 0.5 | 7 + 1  18 + 1  95 + 3  352 + 32 | 19 + 2  102 + 1  256 + 36  3533 + 306 | 20 + 2  38 + 5  73 + 8  158 + 12 | 6 + 0.2  12 + 2  55 + 3  234 + 2 |
| ***ZIKV-MEX*** | 6  24  48  72 | 34 + 3  255 + 21  7561 + 269  8244 + 324 | 0.8 + 0.1  1.1 + 0.4  1.3 + 0.2  1.3 + 0.3 | 5 + 0.2  10 + 1  39 + 1  405 + 15 | 24 + 4  115 + 16  199 + 19  3036 + 483 | 2 + 0.3  6 + 0.2  12 + 0.5  56 + 0.5 | 6 + 0.2  13 + 2  24 + 4  60 + 4 |
| ***DENV-2*** | 6  24  48  72 | 242 + 40  534 + 30  7684 + 126  8613 + 341 | 0.9 + 0.2  1.1 + 0.3  2.8 + 0.2  19 + 0.2 | 19 + 0.2  28 + 0.5  218 + 26  330 + 70 | 58 + 13  198 + 42  2815 + 463  4119 + 631 | 6 + 1  12 + 1  25 + 2  36 + 4 | 266 + 10  272 + 3  527 + 104  1002 + 22 |
| ***YFV-17D*** | 6  24  48  72 | 70 + 6  118 + 19  7300 + 600  8395 + 270 | 0.9 + 0.1  1.3 + 0.1  2.4 + 0.4  20 + 1.9 | 2 + 0.1  15 + 0.3  152 + 7  523 + 13 | 15 + 4  81 + 3  504 + 103  4145 + 235 | 7 + 0.4  13 + 1  1498 + 32  3650 + 450 | 7 + 0.2  11 + 2  19 + 3  451 + 89 |

**Supplemental table. 2: Profile of cytokines, interferons and chemokines secreted by M2-MØ infected with the different flaviviruses.** The supernatants of each condition were collected at different times (6, 24, 48 and 72 hpi). Each value represents the mean concentrations (pg/mL) + standard deviation of three independent experiments (****p < 0.0001; ***p < 0.0008; **p < 0.0056; *p, < 0.0461; ns, not significant).

| **Condition** | **hpi** | **Cytokines (pg/mL)** | | | | | **Interferons (pg/mL)** | |
| --- | --- | --- | --- | --- | --- | --- | --- | --- |
|  |  | **IL-1β** | **IL-6** | **IL-10** | **IL-15** | **TNFα** | **INF-2α** | **INF-*y*** |
| ***MOCK*** | 6  24  48  72 | 49 + 7  103 + 5  204 + 11  300 + 2 | 3 + 0.2  9 + 1  20 + 1  34 + 2 | 21 + 1  52 + 4  89 + 7  123 + 23 | 0.75 + 0.06  1.10 + 0.11  2.50 + 0.10  3.38 + 0.13 | 115 + 17  389 + 18  766 + 30  910 + 7 | 0.4 + 0.1  1.4 + 0.1  3 + 0.1  4 + 0.6 | 0.9 + 0.4  0.9 + 0.4  1.1 + 0.2  1.2 + 0.2 |
| ***ZIKV-MR77*** | 6  24  48  72 | 68 + 3  154 + 9  433 + 74  1125 + 137 | 12 + 2  19 + 2  35 + 5  71 + 2 | 155 + 27  332 + 74  438 + 48  1029 + 16 | 4 + 0.3  6 + 0.4  13 + 0.2  23 + 0.4 | 387 + 19  581 + 36  3998 + 82  5338 + 282 | 21 + 0.5  21 + 0.3  22 + 0.3  22 + 0.9 | 1.4 + 0.4  3.2 + 0.2  3.4 + 0.2  3.8 + 0.3 |
| ***ZIKV-MEX*** | 6  24  48  72 | 24 + 3  79 + 18  222 + 10  417 + 8 | 6 + 1  18 + 2  26 + 3  48 + 2 | 28 + 1  183 + 62  415 + 40  762 + 94 | 3.4 + 1  6.4 + 1  11 + 0.3  20 + 1 | 311 + 85  676 + 139  4025 + 984  5350 + 450 | 21 + 0.2  31 + 0.7  31 + 1  32 + 2 | 1.3 + 0.1  1.8 + 0.1  2.2 + 0.1  2.2 + 0.1 |
| ***DENV-2*** | 6  24  48  72 | 22 + 1  89 + 8  235 + 9  624 + 32 | 10 + 0.2  27 + 1  48 + 2  72 + 4 | 30 + 5  131 + 5  340 + 10  415 + 35 | 7 + 1  12 + 2  17 + 1  22 + 0.5 | 90 + 7  125 + 7  537 + 106  5690 + 451 | 57 + 1  61 + 1  76 + 0.5  129 + 3 | 4.1 + 0.1  11 + 0.7  19 + 0.3  25 + 1.7 |
| ***YFV-17D*** | 6  24  48  72 | 186 + 1  248 + 23  404 + 12  583 + 41 | 36 + 4  47 + 2  64 + 0.2  71 + 1 | 197 + 7  310 + 34  357 + 16  833 + 85 | 4 + 0.3  11 + 1  17 + 0.3  21 + 0.4 | 331 + 28  657 + 95  1148 + 188  6445 + 1045 | 48 + 8  65 + 6  68 + 4  93 + 14 | 3.7 + 0.4  15 + 1.5  27 + 1.5  34 + 2 |

Supplemental table 2, cont.

| **Condition** | **hpi** | **Chemokines (pg/mL)** | | | | | |
| --- | --- | --- | --- | --- | --- | --- | --- |
|  |  | **MCP1 / CCL2** | **MIP1α / CCL3** | **MCP3 /CCL7** | **IL-8 / CXCL8** | **IP-10 / CXCL10** | **VEGF** |
| ***MOCK*** | 6  24  48  72 | 3712 + 109  5017.5 + 265  5876.5 + 137  7155 + 1409 | 399 + 8  1119 + 8  1619 + 4  2061 + 9 | 726 + 53  1554.5 + 179.5  2877.5 + 105.5  4566 + 81 | 3236 + 97  4068 + 38  4718 + 113  5437 + 197 | 42 + 5  52 + 4  113 + 3  138 + 10 | 705 + 153  812 + 72  686 + 8  1621 + 51 |
| ***ZIKV-MR77*** | 6  24  48  72 | 5071 + 577  6819 + 241  13076 + 9711  47760 + 7464 | 830 + 40  2110 + 220  2305 + 75  3183 + 88 | 1400 + 100  6822 + 764  8455 + 12  10555 + 685 | 3847 + 202  4900 + 76  6491 + 89  10113 + 303 | 193 + 14  292 + 80  432 + 17  552 + 62 | 1846 + 135  2579 + 191  3923 + 174  5979 + 593 |
| ***ZIKV-MEX*** | 6  24  48  72 | 5581 + 601  7397 + 210  9674 + 149  11550 + 650 | 843 + 53  2129 + 384  2754 + 74  3273 + 81 | 5402 + 1246  6965 + 135  10403 + 628  12695 + 965 | 4691 + 93  4958 + 55  6001 + 551  6848 + 101 | 49 + 3  128 + 7  235 + 11  324 + 12 | 169 + 31  1474 + 3  1926 + 66  4078 + 619 |
| ***DENV-2*** | 6  24  48  72 | 5753 + 368  6342 + 133  9012 + 99  9899 + 48 | 427 + 5  944 + 34  1727 + 10  2403 + 65 | 1558 + 250  1914 + 31  1963 + 27  3581 + 344 | 4781 + 139  5667 + 501  6207 + 419  7502 + 372 | 72 + 6  105 + 1  128 + 21  159 + 1 | 135 + 1  171 + 26  573 + 18  1856 + 58 |
| ***YFV-17D*** | 6  24  48  72 | 4882 + 526  6464 + 695  7911 + 49  9784 + 569 | 1073 + 27  1665 + 11  1995 + 1  2483 + 78 | 1132 + 206  1560 + 121  2694 + 1077  5827 + 1736 | 3255 + 95  3737 + 47  4003 + 1  5787 + 257 | 186 + 50  298 + 14  353 + 34  413 + 4 | 1651 + 108  1890 + 10  2186 + 155  3751 + 711 |
